# A-NGR fusion protein induces apoptosis in human cancer cells

**DOI:** 10.17179/excli2018-1120

**Published:** 2018-06-25

**Authors:** Azadeh Mohammadi-Farsani, Mehryar Habibi-Roudkenar, Majid Golkar, Mohammad Ali Shokrgozar, Ali Jahanian-Najafabadi, Hossein KhanAhmad, Samira Valiyari, Saeid Bouzari

**Affiliations:** 1 Department of Molecular Biology, Pasteur Institute of Iran, Tehran, Iran; 2 Medical Biotechnology Department, Paramedicine Faculty, Guilan University of Medical Sciences, Rasht, Iran; 3 Molecular Parasitology Laboratory, Department of Parasitology, Pasteur Institute of Iran, Tehran, Iran; 4 National cell bank, Pasteur Institute of Iran, Tehran, Iran; 5 Department of Pharmaceutical Biotechnology, School of Pharmacy and Pharmaceutical Sciences, Isfahan University of Medical Sciences, Isfahan, Iran; 6 Department of Genetics and Molecular Biology, School of Medicine, Isfahan University of Medical Sciences, Isfahan, Iran

**Keywords:** Shiga toxin, NGR peptide, apoptosis, cytotoxicity

## Abstract

The NGR peptide is one of the well-known peptides for targeting tumor cells. It has the ability to target aminopeptidase N (CD13) on tumor cells or the tumor vascular endothelium. In this study, the NGR peptide was used for targeting A subunit of the Shiga toxin to cancer cells. The cytotoxic effect of the A-NGR fusion protein was assessed on HT1080, U937, HT29 cancer cells and MRC-5 normal cells. For this purpose, cells were treated with different concentrations of A-NGR (0.5-40 µg/ml). The evaluation of cell viability was achieved by MTT assay. Apoptosis was determined by annexin-V/PI double staining flow cytometry. Alterations in the mRNA expression of apoptosis - related genes were assessed by real time RT- PCR. The results showed that A-NGR fusion protein effectively inhibited the growth of HT1080 and U937 cancer cells in comparison to negative control (PBS) but for CD13-negative HT-29 cancer cells, only at high concentrations of fusion protein was inhibited growth recorded. On the other hand, A-NGR had little cytotoxic effect on MRC-5 normal cells. The flow cytometry results showed that A-NGR induces apoptosis. Furthermore, the results of real time RT-PCR revealed that A-NGR significantly increases the mRNA expression of caspase 3 and caspase 9. Conclusively, A-NGR fusion protein has the ability of targeting CD13-positive cancer cells, the cytotoxic effect on CD13-positive cancer cells as well as has low cytotoxic effect on normal cells.

## Introduction

Cancer has high mortality and morbidity rates and is regarded as one of the most important health problems around the world (Valiyari et al., 2017[26]). The anticancer drug function is very important in cancer treatment, such that the drug preferentially kills cancer cells without any significant toxic effect on normal cells (Boohaker et al., 2012[2]).

Tumor cells are identified from normal cells, based on expression for the number of molecules on their surface. Hence, a lot of effort was put into distinguishing these surface molecules and also, the corresponding targeting ligands such as peptides, antibody etc. (Di Matteo et al., 2006[10]; Li and Cho, 2012[17]). Therapeutic peptides are a new approach of cancer treatment and are classified into three groups consisting of antimicrobial/pore forming peptides, cell penetration peptide and tumor-targeting peptides (TTPs). TTPs can target markers on the tumor cell membrane such as receptors and can be used to target the delivery of cytotoxic agents to tumor cells or vasculature (Marqus et al., 2017[18]; Thundimadathil, 2012[25]).

One of the most important types of TTPs is NGR (Asn-Gly-Arg) (Marqus et al., 2017[18]). NGR was discovered through the use of *in vivo* phage display technology. It can recognize aminopeptidase N (APN) or CD13 which was expressed in both normal cells and tumor cells. There are several isoforms of APN/ CD13 in different cells and organs. However, studies have shown that only one isoform of CD13 was expressed in tumor cells involved in tumor cells invasion and metastasis (Curnis et al., 2002[7]; Wang et al., 2011[27]).

The NGR peptide is capable of recognizing the tumor-specific isoform of CD13. Furthermore, the NGR peptide can be converted to isoaspartate-glycine-arginine by deamidation of asparagine which is capable of recognizing α_Ѵ_β_3_ integrin. The α_Ѵ_β_3_ integrin is another regulated biomarker in the endothelial cells of angiogenic vessels (Corti et al., 2008[5]; Boohaker et al., 2012[2]; Wang et al., 2011[27]). In regard to the ability of NGR to recognize the tumor specific isoform of CD13 and also α_Ѵ_β_3_, many studies have used NGR for carrying cytotoxic drugs such as DOX, anti-angiogenic drugs ((KLAKLAK)_2 _and endostatin), cytokines (INF-γ,TNF-α) and probe to tumor tissues (Bouchet et al., 2016[3]; Corti, 2004[4]; Curnis et al., 2005[8], 2000[9]; Ellerby et al., 1999[11]; Garde et al., 2007[13]; Meng et al., 2007[20]; Sacchi et al., 2006[23]).

Shiga toxin and Shiga like toxin are produced by *Shigella dysenteriae *and in some serogroups of *Escherichia coli*. These toxins have two subunits consisting of A subunit and a pentamer of five identical B subunits. A subunit is responsible for cytotoxicity while B subunit has the ability of targeting toxins to cells via the globotriaosylceramide (GB3) receptor. A subunit was cleaved into fragments A1 and A2 after internalizing in the cell. The A1 fragment modifies the ribosomal 28SrRNA, inhibits protein synthesis, and initiates apoptosis (Bergan et al., 2012[1]; Engedal et al., 2011[12]; Melton-Celsa, 2014[19]). As a result of the full length A subunit and its truncated forms have a cytotoxic effect, several studies have been conducted on the fusion of A subunit and its truncated forms with a specific targeting moiety (Hotz et al., 2010[14]; Roudkenar et al., 2006[22]).

We constructed, cloned, and expressed the A-NGR fusion protein which consists of A subunit of the Shiga toxin and NGR peptide in our recent study (Mohammadi-Farsani et al., 2017[21]). The effect of this fusion was assessed on U937 cancer cells and the normal cell MRC5 at 48 h. Regarding the cytotoxic effect of A-NGR on U937 and little effect on the viability of MRC5 cells, a decision was made to perform more *in vitro* studies. In this study, the anticancer effect of the A-NGR fusion protein was assessed on HT1080 (CD13-positive cell) and HT-29 (CD13-negative cell) cancer cells. Furthermore, more assessments were done on U937 cancer cells and the MRC5 normal cell at other times.

## Materials and Methods

### Cell culture 

The human cell lines HT1080 (fibrosarcoma), HT-29 (colorectal adenocarcinoma) and MRC-5 (fetal lung fibroblast) were obtained from the Iranian Biological Resource Center (IBRC). U937 (Acute Myeloid Leukemia) was obtained from the Cell bank of Pasteur Institute of Iran (NCBI). MRC-5 and HT1080 were cultured in DMEM/F12 medium, HT-29 was cultured in DMEM medium, and U937 was cultured in RPMI medium. All the media were supplemented with 10 % FBS, 100 U/ml penicillin and 100 µg/ml streptomycin. Cells were incubated at 37 °C and 5 % CO_2_.

### Expression of A-NGR fusion protein 

A-NGR fusion was produced in our recent study (Mohammadi-Farsani et al., 2017[21]). A-NGR (A-GNGRAHA) fusion was constructed by PCR and cloned in pBAD/gIII A vector and expressed in *E. coli *by induction with arabinose. Thereafter, it was purified with Ni-NTA column under denaturing condition. The confirmation of expression was done using SDS-PAGE and western blotting.

### Cell viability assay

The effect of A-NGR fusion protein on cell lines was determined using 3-(4,5-dimethylthiazol-2-yl)-2,5-diphenyltetrazolium bromide (MTT assay), (cell proliferation kit (MTT), Roche, Germany). The MTT assay is a colorimetric assay in which living cells reduce MTT into insoluble formazan while dead cells lose their ability to convert MTT into purple formazan. Briefly, cells were seeded into 96 well plates. A-NGR fusion protein was added at different concentrations ranging from 0.5-40 µg/ml and incubated for 48 and 72 h. Thereafter, the cells were incubated with 10 µl of 5 mg/ml MTT solution for 4 h at 37 °C, then with 100 µl solubilization buffer overnight for dissolving the formazan crystal. The absorbance was measured at 570 nm.

### Identification of apoptosis using Annexin-V/PI staining

The analysis of cell apoptosis was performed using the Annexin-V-FLUOS Staining Kit according to the manufacturer's instructions (Roche, Germany). In brief, HT1080 cells were treated with 72 h-½ IC_50_ and 72 h-IC_50_ of the A-NGR fusion protein. After 48 h incubation, the cells were trypsinized, centrifuged and washed with PBS. Then, the collected cells were resuspended in 100 µl of incubation buffer containing 2 µl Annexin-V-FLOUS labeling reagent and 2 µl PI solution. The cells were incubated at 37 °C, at room temperature for 15 min in the dark. Finally, the cells were analyzed by flow cytometry.

### Real time PCR

Real time RT-PCR was used to assess the changes in mRNA expression of some apoptosis-related genes consisting of caspase 9, caspase 3, and caspase 8. HT1080 cells were treated with 48 h-IC_50_ of A-NGR fusion protein (test cells) and PBS (control cells) for 24 h. The total RNA extraction was done using the NucleoSpin RNA kit (MN, Germany). The concentration and purity of extracted RNA was determined by measuring the absorbance. 1 µg of the extracted RNA was used to perform reverse transcription by the Revert Aid First Strand cDNA Synthesis Kit (Thermo Lithuania). GAPDH was preserved as a reference gene. Real time PCR was done using Human Apoptosis RT Array ^TM^kit (NanoCinna, Iran) in a reaction volume of 15 µl including 2 µl cDNA under condition 95 °C 10 min followed by 50 cycles at 95 °C 25 s, 58 °C 25 s, 72 °C 15 s and melt at 67-95 °C.

### Statistical analysis 

One-way ANOVA was used to determine the differences among groups. The results were expressed as mean ± SD. IC_50_ values were calculated by non-linear-regression analysis with Graph Pad Prism 5 software. P value< 0.05 was considered to be significant.

## Results

### Evaluation of cell viability 

**Figure 1:**
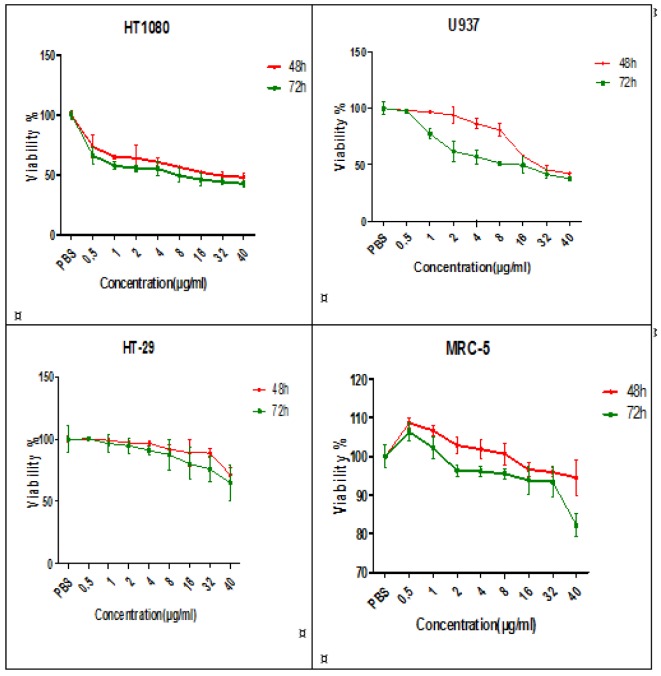
The effect of A-NGR fusion protein on cell viability of HT1080, U937, HT-29 and MRC-5 cells at 48 h and 72 h. The data are presented as mean ± SD of three independent experiments.

The effect of A-NGR fusion protein was assessed on HT1080, HT29, U937 and MRC-5 cells using MTT assay at 48 and 72 h. For HT1080 and U937, as CD13 receptor positive cancer cells were observed a significant decrease in viability at 48, and 72 h compared to the control cells (PBS-treated cells). 48 h-IC_50_ of A-NGR for HT1080 and U937 were determined as 26.21 µg/ml and 26.86 µg/ml, respectively. The HT-29, as a CD13 receptor negative cancer cell, had a significant effect on viability only at high concentrations of the A-NGR fusion protein. The MRC-5 cells, as a normal cell, showed little cytotoxic effect on the viability. These results showed that the A-NGR fusion protein induced apoptosis of HT1080 and U937 cells in a dose- and time-dependent manner (Figure 1(Fig. 1)).

### Identification of apoptosis by Annexin-V/PI staining

**Figure 2:**
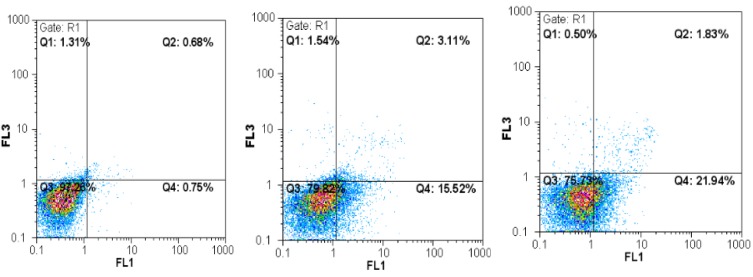
Flow cytometric analysis of annexin-V/PI to quantify A-NGR induced apoptosis in HT1080 cells. a) Dot plot of HT1080 cells treated with PBS. b) Dot plot of HT1080 cells treated with 72 h-½ IC_50_ concentration of A-NGR for 48 h. c) Dot plot of HT1080 cells treated with 72 h - IC_50_ concentration of A-NGR for 48 h

**Figure 3:**
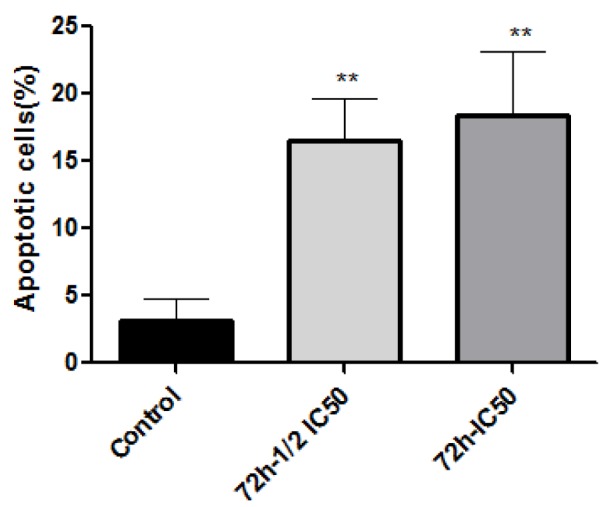
A-NGR fusion protein-induced apoptosis. **P<0.01

The type of cell death induced by A-NGR fusion protein in HT1080 cells was determined by flow cytometry using Annexin V-PI staining, at 72 h-½ IC_50_ and 72 h-IC_50_ concentrations of A-NGR for 48 h. As shown in Figure 2(Fig. 2), the lower left quadrant shows the percentage of live cells (annexin V-/PI-), the lower right depicts the percentage of early apoptotic cells (annexin V+/PI-), the upper right shows late apoptotic cells (annexin V+/PI+), the upper left depicts necrotic cells (annexin V-/PI+). The overall percentage of apoptotic cells are in the lower and upper right quadrants. The treatment of cells with 72 h-½ IC_50_ and 72 h-IC_50_ concentrations of A-NGR fusion protein for 48 h induced 16.49 % and 18.45 % apoptosis (early and late apoptosis) compared with 3.14 % at the control group, respectively. These results showed that the A-NGR fusion protein induces apoptosis in HT1080 cells (Figure 3(Fig. 3)).

### Real time RT-PCR

**Figure 4:**
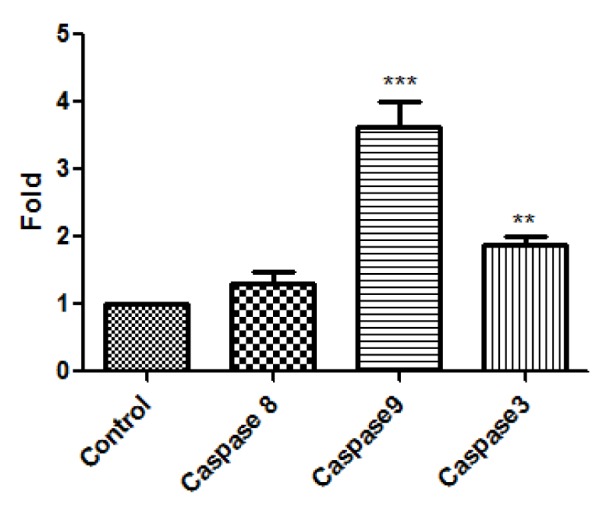
The mRNA expression of caspase 8, caspase 9, and caspase 3 in HT1080 cells was assessed by real time PCR. The data are presented as mean ± SD of three independent experiments. **P<0.01, *** P<0.001

The mRNA expression of caspase 3, caspase 9 and caspase 8 after the treatment of HT1080 cells with A-NGR fusion protein for 24 h was analyzed by Real time RT-PCR. The change of mRNA expression was normalized by GAPDH expression. As shown in Figure 4(Fig. 4), treated cells showed significantly increased mRNA expression of caspase 9 and caspase 3 compared with the control.

### GenBank accession number:

The sequence of A-NGR was submitted in GenBank under accession number MG049787.

See also the Supplementary data.

## Discussion

The NGR peptide should be taken into consideration because of its tumor selectivity property. The receptor of NGR is aminopeptidase N (APN) or CD13. The NGR peptide binds the CD13 isoform expressed in tumor cells and not in other isoforms in normal cells (Curnis et al., 2002[7]). Hence, CD13 is one of the best options for selective drug delivery. For this property, NGR-based drug delivery has been developed in the last decades. Up to now, different compounds such as DOX, TNF, INFγ, tTF have been fused to the NGR peptide. The results of previous studies showed the improvement of anticancer properties of the corresponding compounds (Corti, 2004[4]; Curnis et al., 2005[8], 2000[9]; Ellerby et al., 1999[11]; Garde et al., 2007[13]; Meng et al., 2007[20]; Sacchi et al., 2006[23]). TNF-NGR is the most important of NGR compounds which has recently been tested in phase II and III clinical trials (Corti et al., 2013[6]).

The A-NGR fusion protein has been expressed in *E. coli* (Mohammadi-Farsani et al., 2017[21]). The NGR peptide was used for targeting A subunit of the Shiga toxin to cancer cells. The present study demonstrated that the A-NGR fusion protein could inhibit the growth of CD13-positive HT1080 and U937 cells but showed little cytotoxic effect on CD13-negative HT-29 cells, except at high concentrations that can be because of non-specific toxicity. The A-NGR fusion protein showed little cytotoxic effect on the MRC-5 normal cell. It has been suggested that A-NGR acts via the CD13 receptor and finally results in cell death.

Previous studies were assessed cytotoxic property of Shiga toxin A subunit and catalytic domain of Shiga toxin (A1) when fused to a specific targeting moiety such as GMCSF and VEGF (Hotz et al., 2010[14]; Roudkenar et al., 2006[22]). The A1-GMCSF effect was evaluated on different cell lines such as U937 (Roudkenar et al., 2006[22]). The results of the present study showed that the IC_50_ for A-NGR fusion was higher than A1-GMCSF against U937 (Roudkenar et al., 2006[22]; Jahanian-Najafabadi et al., 2012[15]). It may be due to the use of the full length of subunit A instead of the catalytic domain of Shiga toxin (A1).

Annexin V/PI are widely used to determine live cells, apoptotic and necrotic cells. The evaluation of apoptosis by annexin V-FITC staining showed that the A-NGR fusion protein causes the apoptosis of HT1080 cells compared to the control group.

The mechanism of apoptosis is very complex, there are two main pathways of apoptosis. The extrinsic or death receptor pathway is activated when the death receptor Fas, TNFR1 and death receptor (DR)4/DR5 interact with the death ligands FasL, TNF-α or TRAIL respectively, then caspase 8 is activated which in turn causes the activation of downstream caspases. The intrinsic or mitochondria pathway is activated followed by UV light or cytotoxic chemotherapeutic drugs or oxidative stress that resulted to the formation of pores in the mitochondrial membrane and the release of cytochrome C, then caspase 9 was activated which in turn resulted to the activation of caspase 3 and finally, apoptosis (Tesh, 2010[24]; Wong, 2011[28]).

The results of the present study showed that A-NGR fusion significantly increased the mRNA expression of caspase 9 and caspase 3 compared with the control in HT1080 cells. Previous studies showed that stx induce apoptosis in many epithelial and endothelial cells by activating both the extrinsic and intrinsic pathways (Tesh, 2010[24]). It has been shown that apoptosis is induced by stx in THP-1 cells, independently of TNFR/Fas (Lee et al., 2005[16]). 

For anti-cancer treatment, it is important that cancer cells are killed without damage to normal cells and surrounding tissues. In this study, A-NGR selectively targeted CD13-positive cancer cells, induced apoptosis in these cells while having little cytotoxic effect on normal cells.

## Acknowledgements

We would like to thank Pasteur Institute of Iran for financial support of this project.

## Conflict of interest

The authors declare that they have no conflict of interest.

## Supplementary Material

Supplementary data
