# Supplementary material for: A-NGR fusion protein induces apoptosis in human cancer cells
Source: EXCLI J. 2018 Jun 25;17:590–7. doi: 10.17179/excli2018-1120 (PMC6088213; doi:10.17179/excli2018-1120)
Supplement: Supplementary data [file EXCLI-17-590-s-001.pdf]

EXCLI Journal 2018;17:590-597 – ISSN 1611-2156
Received: February 04, 2018, accepted: May 04, 2018, published: June 25, 2018

Supplementary data to:

A-NGR FUSION PROTEIN INDUCES APOPTOSIS IN HUMAN CANCER CELLS

Azadeh Mohammadi-Farsani[1], Mehryar Habibi-Roudkenar[2], Majid Golkar[3], Mohammad Ali Shokrgozar[4], Ali Jahanian-Najafabadi[5], Hossein KhanAhmad[6], Samira Valiyari[1], Saeid Bouzari[1,*]

[1] Department of Molecular Biology, Pasteur Institute of Iran, Tehran, Iran
[2] Medical Biotechnology Department, Paramedicine Faculty, Guilan University of Medical Sciences, Rasht, Iran
[3] Molecular Parasitology Laboratory, Department of Parasitology, Pasteur Institute of Iran, Tehran, Iran
[4] National cell bank, Pasteur Institute of Iran, Tehran, Iran
[5] Department of Pharmaceutical Biotechnology, School of Pharmacy and Pharmaceutical Sciences, Isfahan University of Medical Sciences, Isfahan, Iran
[6] Department of Genetics and Molecular Biology, School of Medicine, Isfahan University of Medical Sciences, Isfahan, Iran

* Corresponding author: saeidbouzari@yahoo.com

http://dx.doi.org/10.17179/excli2018-1120

This is an Open Access article distributed under the terms of the Creative Commons Attribution License (http://creativecommons.org/licenses/by/4.0/).

Percentage of cell viability after treatment with A-NGR fusion protein for 48 and 72 h

Table 1: Viability of HT1080 cells after treatment with A-NGR fusion protein for 48 h

Concentration	Percentage of cell viability		
0.5 µg/ml	67.33	69.14	84.72
1 µg/ml	63.16	64.83	66.50
2 µg/ml	53.56	64.41	74.43
4 µg/ml	60.52	64.27	57.60
8 µg/ml	56.07	57.73	55.93
16 µg/ml	51.06	53.84	52.03
32 µg/ml	46.19	48.69	52.73
40 µg/ml	50.92	44.52	48.69

EXCLI Journal 2018;17:590-597 – ISSN 1611-2156
Received: February 04, 2018, accepted: May 04, 2018, published: June 25, 2018

Table 2: Viability of HT1080 cells after treatment with A-NGR fusion protein for 72 h

Concentration	Percentage of cell viability		
0.5 µg/ml	62.37	62.20	73.84
1 µg/ml	61.39	55.18	56.67
2 µg/ml	57.54	56.67	52.42
4 µg/ml	58.50	58.17	48.38
8 µg/ml	56.15	45.24	47.05
16 µg/ml	40.09	47.72	50.27
32 µg/ml	46.32	45.27	41.15
40 µg/ml	43.52	39.64	45.02

Table 3: Viability of U937 cells after treatment with A-NGR fusion protein for 48 h

Concentration	Percentage of cell viability		
0.5 µg/ml	98.04	98.01	97.95
1 µg/ml	97.06	96.21	97.03
2 µg/ml	103.04	90.29	88.92
4 µg/ml	90.49	87.65	81.27
8 µg/ml	87.23	77.75	77.75
16 µg/ml	58.82	57.35	56.96
32 µg/ml	49.08	46.43	41.23
40 µg/ml	40.99	43.54	42.56

Table 4: Viability of U937 cells after treatment with A-NGR fusion protein for 72 h

Concentration	Percentage of cell viability		
0.5 µg/ml	96.41	98.66	97.66
1 µg/ml	77.46	73.09	81.84
2 µg/ml	56.73	56.50	72.65
4 µg/ml	63.90	51.57	56.28
8 µg/ml	50.67	51.79	51.23
16 µg/ml	46.64	57.85	45.07
32 µg/ml	38.52	45.91	40.09
40 µg/ml	38.64	38.19	36.39

Table 5: Viability of HT-29 cells after treatment with A-NGR fusion protein for 48 h

Concentration	Percentage of cell viability		
0.5 µg/ml	100.82	99.02	99.65
1 µg/ml	99.74	99.40	98.54
2 µg/ml	99.41	92.82	99.20
4 µg/ml	97.99	94.36	97.99
8 µg/ml	87.91	96.19	91.39
16 µg/ml	78.44	100.11	89.28
32 µg/ml	93.32	88.22	85.42
40 µg/ml	77.70	65.89	70.74

EXCLI Journal 2018;17:590-597 – ISSN 1611-2156
Received: February 04, 2018, accepted: May 04, 2018, published: June 25, 2018

Table 6: Viability of HT-29 cells after treatment with A-NGR fusion protein for 72 h

Concentration	Percentage of cell viability		
0.5 µg/ml	100.43	100.48	100.38
1 µg/ml	92.09	93.13	104.59
2 µg/ml	95.82	100.72	88.38
4 µg/ml	88.53	94.62	90.11
8 µg/ml	93.30	73.58	95.72
16 µg/ml	93.01	67.39	80.58
32 µg/ml	87.88	69.45	70.70
40 µg/ml	80.87	59.56	54.47

Table 7: Viability of MRC-5 cells after treatment with A-NGR fusion protein for 48 h

Concentration	Percentage of cell viability		
0.5 µg/ml	109.09	109.70	107.07
1 µg/ml	106.01	108.27	105.81
2 µg/ml	100.50	103.07	105.05
4 µg/ml	99.17	102.90	103.53
8 µg/ml	97.74	100.77	103.37
16 µg/ml	95.98	95.27	98.59
32 µg/ml	96.87	94.56	96.36
40 µg/ml	90.09	94.27	99.12

Table 8: Viability of MRC-5 cells after treatment with A-NGR fusion protein for 72 h

Concentration	Percentage of cell viability		
0.5 µg/ml	104.78	108.85	105.25
1 µg/ml	105.22	101.05	100.24
2 µg/ml	97.82	94.85	96.41
4 µg/ml	97.65	95.51	95.25
8 µg/ml	96.55	95.86	94.00
16 µg/ml	93.36	97.47	90.56
32 µg/ml	89.51	97.25	93.61
40 µg/ml	85.67	80.13	80.79

Data was analyzed with Graph Pad Prism 5 software.

EXCLI Journal 2018;17:590-597 – ISSN 1611-2156
Received: February 04, 2018, accepted: May 04, 2018, published: June 25, 2018

Table 9: Identification of apoptosis by annexin-V/PI staining

Treatment	The percentage of early and late apoptotic cells after treatment		
Control	1.43	4.52	3.48
72 h-1/2IC_{50}	12.83	18.02	18.63
72 h-IC_{50}	14.95	23.77	16.64

Control: The percentage of early and late apoptotic cells after treatment with PBS.
72 h-½ IC_{50}: The percentage of early and late apoptotic cells after treatment with A-NGR fusion protein at 72 h-½ IC_{50} concentration for 48 h.
72 h-IC_{50}: The percentage of early and late apoptotic cells after treatment with A-NGR fusion protein at 72 h-IC_{50} concentration for 48 h.

Table 10: The analysis of mRNA expression of caspase 8, caspase 9 and caspase 3 by real time RT-PCR

	Fold change		
**Caspase 8**	1.49	1.24	1.15
**Caspase 9**	3.39	3.46	4.06
**Caspase 3**	1.77	1.91	1.99

The relative expression of each gene was analyzed by comparative threshold cycle (Ct). Ct value was normalized using the formula ΔCt = Ct (apoptotic genes) - Ct (GAPDH). Then formula ΔΔCt = ΔCt (treated) - ΔCt (control) was used. Finally, the formula 2−ΔΔCt was used for estimating relative expression of each gene.

Figure 1 was extracted from Tables 1 to 8.

Figure 2 is a representative of percentage of Vital cells, Early apoptosis and Late apoptosis when treated with PBS, A-NGR fusion protein.

Figure 3 was extracted from Table 9.

Figure 4 was extracted from Table 10.
